# Tetrahydrocannabinol and dopamine D1 receptor

**DOI:** 10.3389/fnins.2024.1360205

**Published:** 2024-02-14

**Authors:** Jiwon Lee

**Affiliations:** Department of Psychology, Harvard University, Cambridge, MA, United States

**Keywords:** dopamine, THC, D1 receptor, molecular pathway, behavior & cognition

## Abstract

Dopamine, a neurotransmitter and neuromodulator, is primarily released by dopaminergic neurons in the midbrain, particularly in the substantia nigra and the ventral tegmental area (VTA). Dopamine is known to have 5 receptors which are D1, D2, D3, D4 and D5, which are further categorized into 2 families: D1 family and D2 family. The D1 family and D2 family work in conjunction, playing interconnected roles in reward processing and decision-making. The D1 family is composed of D1 and D5 receptors and primarily functions in motivation and motor control. In contrast, the D2 family, composed of D2, D3, and D4 receptors, affect attention and sleep. THC, a type of cannabinoid, can lead to feelings of euphoria, anxiety, fear, distrust, or panic, and modulates dopamine activity in several regions of the central nervous system. Although there is a vast amount of research between the relationship of THC on dopamine, there continues to be limited research in relation to THC on dopamine receptors. The D1 receptor plays a role in several essential functions, such as memory, attention, impulse control, regulation of renal function, and locomotion. Accordingly, this review is intended to summarize the relationship between THC and D1 receptors, highlighting key gaps in the literature and avenues for future research.

## 1 Introduction

Cannabis is gaining popularity again as a result of the legalization of marijuana. According to a survey of current marijuana use among US residents 12 to 25 years of age during 1979–2016 by National Survey on Drug Use and Health, starting in the 1990s, cannabis use started to increase (Yu et al., 2020).

Cannabinoids are substances that are found in cannabis and can interact with the endocannabinoid system. One of the most well-known cannabinoids is Tetrahydrocannabinol (THC). This substance interacts with the endocannabinoid system and affects different parts of the body. This system contains two receptors: CB1 and CB2. CB1 receptors are located in different parts of the CNS, including the cerebral cortex, amygdala, and hippocampus, and are associated with processes related to memory, learning, motor skills, and emotional responses (Busquets Garcia et al., 2016). In contrast, CB2 receptors are primarily found in the peripheral nervous system (PNS), particularly in immune cells such as those in the spleen and macrophages, but they can also be present in the CNS (including the brainstem and CA2/3 pyramidal neurons of the hippocampus) and play a role in regulating immune functions (Turcotte et al., 2016). THC affects dopamine, a neurotransmitter involved in motor control, arousal, and more (Gunasekera et al., 2021). Specifically, within the central nervous system, THC regulates dopaminergic activity across several regions, including the dorsal and ventral striatum. Additionally, studies suggest that THC influences the anterior cingulate cortex (ACC), where dopamine plays a crucial role in regulating associated functions. This indicates that the effects of THC on the ACC may be mediated by its impact on dopamine signaling (Khani et al., 2014; Borgwardt et al., 2008; Bloomfield et al., 2016).

Dopamine is a monoamine neurotransmitter, which plays a role in executive function, motor control, motivation, arousal, reinforcement, and reward. Central dopamine release can be stimulated by motivating stimuli and engaging in rewarding behaviors, such as nucleus accumbens. While dopamine is primarily produced in the brain by neurons in the substantia nigra and ventral tegmental area, the adrenal glands can also release dopamine into the bloodstream under certain conditions, such as stress. This causes the adrenal gland to release dopamine. There are four major dopaminergic pathways, the mesocortical pathway, the nigrostriatal pathway, the tuberoinfundibular pathway and the mesolimbic pathway (Braverman, 2024). Dopamine has a number of essential functions in the brain. In particular, dopamine released from the midbrain plays a crucial role in the mammalian central nervous system. Dopamine has a total of 5 receptors which are divided into two main functional categories (Bhatia and Saadabadi, 2020).

Although a great deal of research has been conducted on the effect of THC on dopamine, the amount of research conducted on the influence of THC on dopamine receptors is limited. Therefore, this review aims to elucidate the relationship between THC and D1 receptors.

## 2 Dopamine

Dopamine is a neurotransmitter and neuromodulator, which is synthesized in the ventral tegmental area (VTA), substantia nigra, and hypothalamus. It has a catechol structure (a benzene ring with two hydroxyl side groups) (Kaufman, 2007; Oyama, 1973) with one amine group attached via an ethyl chain. Dopamine is part of the catecholamine family, which is a type of a neurotransmitter composed of catechol and a side-chain amine. The process of dopamine synthesis starts with the amino acid phenylalanine going through several processing steps, involving tyrosine and DOPA (dihydroxyphenylalanine) as intermediates before finally forming dopamine (National Center for Complementary and Integrative Health, 2019; Harsing, 2008).

### 2.1 CB1 receptor

The CB1 receptor is known to be part of the endocannabinoid system, which modulates activity in the brain, endocrine system, immune cells, and other parts of the body. Additionally, it is involved in stress responses and reproductive functions. The CB1 receptor is known to be a G protein-coupled receptor (GPCR), a type of receptor that is connected to heterotrimeric G proteins. G proteins are membrane proteins essential for transmitting extracellular signals into intracellular responses (Howlett et al., 2010; Zou and Kumar, 2018).

Dopaminergic pathways have many GABA(ergic) terminals which are modulated by CB1 receptors and endocannabinoids (such as anandamide and 2-AG). This modulation involves retrograde feedback, which is important for regulating synaptic transmission and maintaining balance between excitatory and inhibitory signals. This interaction also affects dopamine release, especially in areas such as the nucleus accumbens (NAc). When endocannabinoids act on CB1 receptors, they increase dopamine release. This mediates the dopaminergic effects of endocannabinoids, which is shown by CB1 antagonist rimonabant blocking the release of dopamine from the NAc shell that is stimulated by anandamide and 2-AG. Based on synaptic activity, the midbrain’s response is altered, contributing to the rewarding effects of THC. This leads to increased dopamine release and firing of dopamine neurons. 2-AG, once produced, activates CB1 receptors on glutamate and GABA neurons. These receptors reduce GABA’s effect on dopamine cells in the midbrain, which increases dopamine activity. This represents the role of CB1 receptors in dopamine regulation. Additionally, CB1 receptors play a role in balancing the excitatory and inhibitory signals which influence dopamine neurons, which is essential for stabilizing the activity of dopamine. Endocannabinoids modulate retrograde suppression in glutamate terminals, where CB1 receptors are localized and synapse with midbrain dopamine neurons. Therefore, the evidence outlined above indicates a key role of endocannabinoid and CB1 receptors in the dopamine system (Bloomfield et al., 2016).

### 2.2 CB2 receptor

CB2 receptor, similar to CB1 receptor, is part of the endocannabinoid system and is GPCR. CB2 receptor is primarily involved with 2-Arachidonoylglycerol (2-AG) and is mostly expressed in the peripheral immune system (Munro et al., 1993; Basu et al., 2011). However, it can also be seen in CNS, primarily on microglia, but the expression is low in other regions of CNS (Pertwee, 2006).

The CB2 receptor is known to inhibit adenylyl cyclase through Giα/Goα protein subunits, where Gβγ subunits are signaled by mitogen-activated protein kinases (MAPK) and extracellular signal-regulated kinases (ERK) pathways. CB2 receptors are deactivated by G-protein-coupled receptor kinases, which internalize the receptor and facilitate arrestin binding (Yeliseev and Gawrisch, 2017).

Because CB2 receptors are largely involved with 2-AG, an endocannabinoid known to defend the CNS, they mediate anti-inflammatory, anti-apoptotic, and neuroprotective properties. This provides a therapeutic focus for protecting the nervous system in glaucoma patients. Additionally, Magid et al. (2019) have proposed that CB2 receptor agonists HU-910 and HU-914 are potential drugs for developing a new therapeutic approach for traumatic brain injury (Magid et al., 2019; Lindner et al., 2023).

CB2 modulates mitochondrial function, oxidative stress, iron transport, and neuro-inflammation which would otherwise result in neuronal cell death. CB2 receptors provide feedback on electrophysiological processes, pointing to potential new treatments for Parkinson’s disease (Yu et al., 2024).

CB2 receptors are also known to guide behavior. For example, Han et al. (2017) displayed that when the CB2 receptor was selectively deleted from dopamine neurons, there was a subsequent increase in the basal level of locomotor behavior and altered WIN-55212-2-induced analgesia and catalepsy.

## 3 THC and dopamine

THC is one of the most common types of cannabinoids, which is a type of psychoactive that can be found in marijuana (National Academies of Sciences, Engineering, and Medicine, 2017). Its functional groups are the Benzene ring, secondary alcohol, 3 methyl groups, and an oxane ring. It is known to cause problems in senses, moods, motor movement, cognitive ability and with high doses, it can cause hallucinations, delusions and psychosis. Although adults are affected by marijuana, children under the age of 18 are particularly susceptible to THC, since it can interfere with brain development. When consumed as a child, THC can cause problems in brain connectivity and affect attention, memory, problem-solving skills, behavior, and overall cognitive abilities (Volkow et al., 1996).

THC affects cells by binding to cannabinoid receptors. This process leads to an increase of synthesis and release of dopamine. Through looking at changes of the cerebral blood flow and glucose metabolism, functional magnetic resonance imaging (fMRI) and positron emission tomography (PET) can indirectly evaluate dopaminergic function. For humans, acute THC administration is associated with increased activity of the frontal and subcortical region (Pertwee and Ross, 2002). Since the CB1 receptor concentration rate is highest in these areas (Bossong et al., 2015), rather than dopaminergic-mediation, endocannabinoid might be the effect on the result.

Research has shown that it is possible to use molecular imaging to measure activity in the dopamine system. Studies using this method looked at the effects of THC administration on human dopamine release with exposure to cannabis. By examining with PET, two of the previous studies show that dopamine release in the ventral striatum can be caused by THC (Stokes et al., 2010). Similarly, in the fronto-temporal cortical region of the brain, dopamine release was evoked by acute THC challenge (Bloomfield et al., 2016; Anmol et al., n.d.).

## 4 Dopamine receptors

Dopamine is known to have five receptors (D1, D2, D3, D4, D5 receptors) which are divided into 2 families (D1 and D2 like receptor families). D1-like family receptors include D1 and D5 receptors and D2 family receptors include D2, D3 and D4. The D1-like receptor family plays a role in memory, attention, impulse control, regulation of renal function, and locomotion (D1 receptor), as well as decision making, cognition, attention, and renin secretion (D5 receptor). The D2-like family of receptors play a role in locomotion, attention, sleep, memory, and learning (D2 receptor), as well as cognition, impulse control, attention, and sleep (D3, D4 receptor) (Mishra et al., 2018).

The D1-like receptors (D1, D5) are most common in striatum, caudo-putamen, nucleus accumbens, SN pars reticulata, or olfactory bulb. These receptors play a role in the reward system, locomotor activity, learning, and memory. By the activation of guanosine nucleotide-binding proteins (G proteins), D1 receptors foster the simulation of adenylyl cyclase (AC), and also generate cyclic AMP for its secondary messengers. D1 receptors are also linked with neuropsychiatric disorders.

The D2-like receptors (D2R) consist of D2, D3, and D4 receptors. There are two subtypes: D2-short and D2-long. The D2R are mainly located in the striatum, external globus pallidus (GPe), core of NAcc, amygdala, cerebral cortex, hippocampus, and pituitary. The D2R usually inhibits AC activity and hampers cAMP and PKA production levels. Postsynaptic D2 receptors have an essential role in mediating behavioral and extrapyramidal activity (Hasbi et al., 2020).

## 5 D1 receptor

### 5.1 THC effect on D1 receptor density

There have been studies on the relationship of THC and D1 receptors on D1 receptor’s density. In a study conducted with African Green Monkeys, it was found that caudate putamen (CPu), which expresses D1 and D2 heteromer, exhibited a significant increase in the number of neurons compared to the control group. As for nucleus accumbens (NAc), it was also observed that the group using THC showed an increase in the density of D1-D2 heteromers. Specifically, they used a proximity ligation assay to determine D1-D2 heteromer levels, and found that, compared to the control group, the THC group showed a 3-fold increase in positive signal. The research also indicated that, following THC administration, neurons exhibited diversity, with most having approximately 4 fluorescent signals per neuron, while a few displayed as many as 17 signals, whereas others had 2–4 signals per nuclei. However, to know if D1-D2 heterodimerization increase by THC is connected to the D1 or D2 receptor ratio level, RT-PCR was carried out on extracts from caudate-putamen and NAc to determine the mRNA expression levels of D1 and D2. As for D2 or the dopamine transporter DAT, there was no significant effect on mRNA expression, but for the D1 receptor, persistent THC usage up-regulated mRNA expression (Jewett and Thapa, n.d.).

While findings in primates are more applicable to humans, rodents are more amenable to a wider range of experimental manipulations. Indeed, a study used female rats to show that THC increased D1 receptor density in NAc. As for male rats, decreased D1 receptor density in PFC was reverted by adolescent THC. Additionally, maternal deprivation (MD) can induce behavioral responses via D1 and D2 receptors and NMDA receptors, glutamate receptors which play a crucial role in synaptic plasticity (Zamberletti et al., 2012), which can be modulated by THC (Navarro et al., 1993). One study dosed male rats with THC and measured the number and affinity of D1 and D2 receptors in the striatum. They observed a loss of spontaneous motor activity following oral doses of THC. This correlated with a decrease of D1 receptors in the striatum (Navarro et al., 1993). Others have discovered a relationship between THC and D1 receptors such as Komeili et al. (2021), which have found that in male rats with 6-hydroxydopamine-induced substantia nigra lesions, dosing with marijuana extract led to an increase in PSD-95 and D1 receptors.

### 5.2 Age and sex differences in D1 receptor expression & effect of THC

There are several studies that used rats and rodent models to investigate sex differences in D1 receptor expression. Rodent models are commonly used to demonstrate sex differences in D1 receptor expression. A study showed that juvenile male rats compared to juvenile female rats showed greater concentrations of D1 receptors in the striatum and cortex. Additionally, another study has shown that, during puberty, male rats exhibited brief overproduction of dorsal striatal D1 receptors compare to female rats. However, this difference did not persist into adulthood (Andersen et al., 1997; Andersen and Teicher, 2000; Orendain-Jaime et al., 2016). On the other hand, compared to female rats, male rats showed an overproduction of dopamine D1 receptors in NAc which persisted into adulthood. In the infralimbic cortex, females exhibited a higher D1:D2 expression ratio throughout the developmental stage. However, for males, there was a significant rise in the D1:D2 expression ratio in the insular cotex throughout development (Andersen et al., 1997; Cullity et al., 2018; Hasbi et al., 2020).

These sex-dependent variations in D1 receptor expressions lead to different effects of THC. For example, Pérez-Valenzuela et al. (2023) examined biomarkers related to mood and anxiety disorders that include protein kinase B (Akt), glycogen synthase kinase-3 (GSK-3), BDNF, mTOR, D1, and D2 receptor in nucleus accumbens (NAc) and PFC of rats that consumed THC: CDB. It was shown that only stressed male rats, and not stressed female rats, expressed great anxiolytic and antidepressant effects.

Several articles showed age-dependent differences in D1 receptors. For example, Wang et al. (1998) investigated differences in D1 receptor binding using PET with [11C]SCH 23390 in 21 people across a wide range of ages and showed decline of D1 receptor with age. Additionally, another study suggested that in the striatum and frontal cortex, the binding potential (k3/k4) decreased by 35 and 39% with age (Suhara et al., 1991). Lastly, Meng et al. (1999) suggested that D1 receptor expression started at 19 gestational weeks and decreased after 6–8 months. However, given that gender also affected these D1 receptor levels, the researchers did not account for gender in these observations.

Comparison of untreated and THC treated adolescent and adult rats revealed that adult rats exhibit an upregulation in D1-D2 heteromer expression within NAc and dorsal striatum areas of the brain. While adolescent rats did not show any change in expression. Additionally, it was shown that lower striatal densities of D1-D2 heteromer for adolescent rats than adults. The authors attribute this change with the following behaviors in adolescent rats: anxiety and anhedonia-like behavior. Lastly, downstream markers of D1-D2 activation were modified by THC for adults, but not for adolescents striatum (Di Raddo et al., 2023). However, further research should be explored to gain a deeper understanding of these topics.

### 5.3 THC-D1 in behavior & cognition

There are various behavioral aspects that were studied when analyzing the relationship between THC and D1 receptors. For instance, a study using male rodents studied hedonic and aversion-like behaviors as a result of daily THC consumption and its subsequent spontaneous withdrawal. It found that anxiogenic-like and anhedonic-like behaviors, as well as neurochemical changes, were reversed by an interference peptide disrupting D1-D2 heteromer during withdrawal (Hasbi et al., 2023). Additionally, Mani et al. (2001) used female rats to study sexual receptiveness in response to THC. The study found that PR and D1 receptors were needed for the THC response. Additionally, it also suggests that THC-facilitated receptivity involves D1B (D5) receptors. One potential mechanism is that the effects of THC are mediated via CB1 receptors inducing dopamine release and thus stimulating D1 receptors. However, as the study was conducted only on female rats, the generalisability is limited. Therefore, future work should aim to investigate this mechanism in male rats.

D1 and THC are also thought to influence feeding and motor control. Verty et al. (2004) used Wistar rats between the ages of 8–10, and have shown that cannabinoid-induced food ingestion can be involved with D1-like receptors. SCH 23390 was shown to attenuate THC-induced feeding when given at a dose that was insufficient to affect feeding when given in the absence of THC. SCH-23390 also attenuated locomotion affected by THC. In addition, when THC was given with SCH-23390, SCH 23390 did not show suppressive effects on locomotor activity. In further work investigating the role of THC on motor control (de Fonseca et al., 1994), chronically stimulated D1 and D2 receptors in male rats and observed the role of 11-hydroxy-delta 8-tetrahydrocannabinol-dimethylheptyl (HU-210), a cannabinoid agonist which is highly potent. It has been demonstrated that HU-210-induced catalepsy, as measured by descent latency in a bar test, was enhanced in the presence of SKF38393, which is a selective partial agonist of D1/D5 receptor. This result might suggest that D1 receptor plays a role in cannabinoid induced catalepsy (de Fonseca et al., 1994). However, future work should aim to replicate this study in female rats to ensure the effect is not gender-specific.

In contrast to the key roles of THC and D1 in the above behaviors, no role has been observed in discrimination or withdrawal effects. For example, Solinas et al. (2010) used rats to demonstrate that D1 receptors did not augment THC discrimination, which is a type of experiment that tests an animal’s ability to distinguish between THC and control substances. The D1 receptor’s antagonist, SCH-23390, did not play a role in reducing the THC discrimination effect. Additionally, D1 receptor’s antagonist did not counteract THC discrimination yielded by cocaine and amphetamine augmentation. The D1 receptor antagonist also did not show a discriminative effect at low doses of THC. In other work, Sañudo-Peña et al. (1999) treated rats with THC or vehicle and then subsequently SCH23390 sulpiride was administered. Then, their behaviors were investigated for 1 h after getting injected with SR141716A, a selective CB1 antagonist, or vehicle. The study revealed that the cannabinoid triggered a withdrawal syndrome that was not weakened by treatment with dopamine antagonists in THC tolerant animals. However, syndromes were increased by agonists.

There has been research studying the relationship of THC and D1 receptors on cognitive activity. There has been an effect of THC and D1 receptor antagonist interaction affecting cognitive ability. A study suggests that working memory impairment by THC can be prevented by D1-like and D2-like antagonists. This suggests that excessive dopaminergic activation in the medial prefrontal cortex causes impairment of the working memory (Rodrigues et al., 2011).

Interestingly, a study observed D1 and D2-Cre transgenic rats that learned to self-administer THC and cannabidiol and observed a decrease of spine heads in D1 medium spiny neurons occurring after extinction from THC and CBD (Garcia-Keller et al., 2023). While they did not measure the cognitive impact of this spine loss, spine loss is a known correlate of cognitive dysfunction.

### 5.4 THC-D1 molecular pathway

Elucidating the molecular pathways by which THC mediates its effect on D1 receptors could highlight potential molecular targets for therapeutic intervention. Indeed, studies have revealed numerous molecules involved in the THC-D1 molecular pathway. For example, with the application of THC, the expression of dopamine D1-D2 receptor heteromer increased sharply in nucleus accumbens. This was accompanied by a calcium-linked signaling increase and BDNF/TrkB pathway activation, dynorphin expression, and signaling of kappa opioid receptors (Svenningsson et al., 2004). In other studies, the THC-induced phosphorylation of DARPP-32, a protein that responds to physiological and pharmacological stimuli by regulating electrophysiological, transcriptional, and behavioral responses (Borgkvist et al., 2008), was inhibited by SCH-23390. This suggests that D1 receptors are preferentially expressed in a pathway that increases DARPP-32 phosphorylation in neurons by THC. Additionally, an intact D1 receptor and A2A, a receptor that interacts with dopamine receptors and modulates glutamatergic regulation of GABAergic and enkephalergic neurons, receptor is necessary for the action of THC, because phosphorylation of DARPP-32 at Thr34, which is important process for the indirect and direct pathway of striatal neurons, is represented by basal activation of those two receptors (Miyamoto et al., 1996).

The D1 receptor-mediated effects of THC are also thought to rely on Fos expression. For example, Miyamoto et al. (1996) showed that in dorsomedial striatum and nucleus accumbens, SCH 23390 blocked Fos expression induced by THC (Daigle et al., 2011). Similarly, Lazenka et al. (2015) show that before administration of THC, treating with D1 receptor antagonists can attenuate Δ FBJ murine osteosarcoma viral oncogene homolog B (ΔFosB), a protein part of Fos family with a transcription function, in the in the prefrontal cortex, amygdala, caudate-putamen, and nucleus accumbens. Additionally, the study suggests that SCH 23390 inhibited ΔFosB expression that is THC-stimulated, but it induced ΔFosB expression in nucleus accumbens, which might be due to 5-HT2R, a serotonin receptor which modulates monoaminergic transmission, mood, motor behavior, appetite and endocrine secretion (Millan, 2005), activity (Lazenka et al., 2015).

Finally, ERK has also been implicated in the THC-D1 receptor mediated effects. A study that used male CD-1 mice has shown that in striatum administration of acute THC activates ERK. When SCH-23390 was used, it has been shown that the activation of ERK decreased in striatum. This result may indicate that the D1 receptor is involved in THC-stimulated ERK activation (Valjent et al., 2001). Additionally, Daigle et al. (2011) have found that when treated with HU-210, striatal HU-210-dependent ERK1/2 signaling in striatum decreased more than in the wild-type models. The research also suggests that D1 receptors might act as a signal transduction of CB1 cannabinoid in an opposing manner.

## 6 Discussion

As highlighted in this review, there is a complex relationship between THC and the D1 receptor. The modulation of D1 receptors by THC has significant implications for understanding the cognitive and behavioral effects of cannabis. In particular, in the context of increasing cannabis use following its legalization in various regions around the world, it is essential to understand the diverse array of cognitive and behavioral changes associated with THC use and what molecular mechanisms mediate this. As discussed, research on D1 receptor-mediated effects of THC is particularly lacking.

This review has highlighted the diverse role of THC on D1 receptor density and the various behaviors influenced by the THC-D1 receptor pathway, such as feeding, sexual receptiveness and withdrawal effects. In addition, the D1 receptor is essential for mediating at least some of the effects of THC on motor control and cognition. These effects could be enacted via molecular pathways involving a number of molecules, including the BDNF/TrkB pathway, Fos expression, and ERK activation.

However, this body of research has a number of limitations. For instance, there is substantial reliance on animal models, particularly rodents. While these insights are valuable, the generalisability to the human condition is limited. Therefore, it’s important for future studies to bridge the gap between animal and human research by, for example, conducting longitudinal human studies to better understand the long-term effects of THC and the role that D1 receptors play in mediating this. Additionally, some of the studies were focused on only one sex, hence it is possible that sex differences could exist in the D1-receptor mediated effects of THC. Therefore, it is important to conduct studies on both sexes in order to identify any potential sex differences regarding THC and its interaction with the D1 receptor.

Given the susceptibility of the adolescent brain to the effects of THC, it’s crucial for further work to investigate how THC exposure during critical developmental periods impacts cognitive and behavioral outcomes, and the potential role of the D1 receptor in mediating these effects. Lastly, it will be important to bridge the gap between the molecular pathways and the behavioral effects in order to reveal potential molecular targets for therapeutic intervention.

In conclusion, there is a large gap within the knowledge regarding THC and its relation with the D1 receptor. This should be addressed with future research in pharmacology, neuroscience, and medical professionals.

## Author contributions

JL: Investigation, Validation, Writing – original draft, Writing – review & editing.
