# Correction: Tetrahydrocannabinol and dopamine D1 receptor

**DOI:** 10.3389/fnins.2025.1693909

**Published:** 2025-11-10

**Authors:** Jiwon Lee

**Affiliations:** Department of Psychology, Harvard University, Cambridge, MA, United States

**Keywords:** dopamine, THC, D1 receptor, molecular pathway, behavior and cognition

Busquets Garcia, A., Soria-Gomez, E., Bellocchio, L., and Marsicano, G. (2016). Cannabinoid receptor type-1: breaking the dogmas. *F1000Res*. 5:F1000 Faculty Rev-990. doi: 10.12688/f1000research.8245.1 was not cited in the article. The citation has now been inserted in the section **Introduction**, Paragraph 2 and should read:

“CB1 receptors are located in different parts of the CNS, including the cerebral cortex, amygdala, and hippocampus, and are associated with processes related to memory, learning, motor skills, and emotional responses (Busquets Garcia et al., 2016).”

Turcotte, C., Blanchet, M.-R., Laviolette, M. and Flamand, N. (2016). The CB2 receptor and its role as a regulator of inflammation. *Cell. Mol. Life Sci*. 73, 4449–4470. doi: 10.1007/s00018-016-2300-4 was not cited in the article. The citation has now been inserted in the section **Introduction**, Paragraph 2 and should read:

“In contrast, CB2 receptors are primarily found in the peripheral nervous system (PNS), particularly in immune cells such as those in the spleen and macrophages, but they can also be present in the CNS (including the brainstem and CA2/3 pyramidal neurons of the hippocampus) and play a role in regulating immune functions (Turcotte et al., 2016).”

Gunasekera, B., Diederen, K., and Bhattacharyya, S. (2021). Cannabinoids, reward processing, and psychosis. *Psychopharmacology* 239, 1157–1177. doi: 10.1007/s00213-021-05801-2 was not cited in the article. The citation has now been inserted in the section **Introduction**, Paragraph 2 and should read:

“THC affects dopamine, a neurotransmitter involved in motor control, arousal, and more (Gunasekera et al., 2021).”

Khani, A., Kermani, M., Hesam, S., Haghparast, A., Argandoña, E. G., and Rainer, G. (2014). Activation of cannabinoid system in anterior cingulate cortex and orbitofrontal cortex modulates cost-benefit decision making. *Psychopharmacology* 232, 2097–2112. doi: 10.1007/s00213-014-3841-6 was not cited in the article. The citation has now been inserted in the section **Introduction**, Paragraph 2 and should read:

“THC affects dopamine, a neurotransmitter involved in motor control, arousal, and more. Specifically, within the central nervous system, THC regulates dopaminergic activity across several regions, including the dorsal and ventral striatum. Additionally, studies suggest that THC influences the anterior cingulate cortex (ACC), where dopamine plays a crucial role in regulating associated functions. This indicates that the effects of THC on the ACC may be mediated by its impact on dopamine signaling (Khani et al., 2014; Borgwardt et al., 2008; Bloomfield et al., 2016).”

Borgwardt, S. J., Allen, P., Bhattacharyya, S., Fusar-Poli, P., Crippa, J. A., Seal, M. L., et al. (2008). Neural basis of Δ-9-tetrahydrocannabinol and cannabidiol: effects during response inhibition. *Biol. Psychiatry* 64, 966–973. doi: 10.1016/j.biopsych.2008.05.011 was not cited in the article. The citation has now been inserted in the section **Introduction**, Paragraph 2 and should read:

“THC affects dopamine, a neurotransmitter involved in motor control, arousal, and more. Specifically, within the central nervous system, THC regulates dopaminergic activity across several regions, including the dorsal and ventral striatum. Additionally, studies suggest that THC influences the anterior cingulate cortex (ACC), where dopamine plays a crucial role in regulating associated functions. This indicates that the effects of THC on the ACC may be mediated by its impact on dopamine signaling (Khani et al., 2014; Borgwardt et al., 2008; Bloomfield et al., 2016).”

Bloomfield, M. A. P., Ashok, A. H., Volkow, N. D., and Howes, O. D. (2016). The effects of Δ9-tetrahydrocannabinol on the dopamine system. *Nature* 539, 369–377. doi: 10.1038/nature20153 was not cited in the article. The citation has now been inserted in the section **Introduction**, Paragraph 2 and should read:

“THC affects dopamine, a neurotransmitter involved in motor control, arousal, and more. Specifically, within the central nervous system, THC regulates dopaminergic activity across several regions, including the dorsal and ventral striatum. Additionally, studies suggest that THC influences the anterior cingulate cortex (ACC), where dopamine plays a crucial role in regulating associated functions. This indicates that the effects of THC on the ACC may be mediated by its impact on dopamine signaling (Khani et al., 2014; Borgwardt et al., 2008; Bloomfield et al., 2016).”

Bhatia, A., and Saadabadi, A. (2020). *Biochemistry, Dopamine Receptors*. Treasure Island, FL: StatPearls Publishing was not cited in the article. The citation has now been inserted in the section **Introduction**, Paragraph 3 and should read:

“Dopamine has a total of 5 receptors which are divided into two main functional categories (Bhatia and Saadabadi, 2020).”

Oyama, T. (1973). “Catecholamines,” in *Anästhesiologie und Intensivmedizin* (Berlin: Springer), 73–91. doi: 10.1007/978-3-642-65561-6_7 was not cited in the article. The citation has now been inserted in the section **2 Dopamine**, Paragraph 1 and should read:

“It has a catechol structure (a benzene ring with two hydroxyl side groups) (Kaufman, 2007; Oyama, 1973) with one amine group attached via an ethyl chain.”

Harsing, L. G. (2008). “Dopamine and the dopaminergic systems of the brain,” in *Handbook of Neurochemistry and Molecular Neurobiology*, eds. A. Lajtha, and E. S. Vizi (Boston, MA: Springer), 149–170. doi: 10.1007/978-0-387-30382-6_7 was not cited in the article. The citation has now been inserted in the section **2 Dopamine**, Paragraph 1 and should read:

“The process of dopamine synthesis starts with the amino acid phenylalanine going through several processing steps, involving tyrosine and DOPA (dihydroxyphenylalanine) as intermediates before finally forming dopamine (National Center for Complementary and Integrative Health, 2019; Harsing, 2008).”

Zou, S., and Kumar, U. (2018). Cannabinoid receptors and the endocannabinoid system: signaling and function in the central nervous system. *Int. J. Mol. Sci*. 19:833. doi: 10.3390/ijms19030833 was not cited in the article. The citation has now been inserted in the section **2.1 CB1 Receptor**, Paragraph 1 and should read:

“G proteins are membrane proteins essential for transmitting extracellular signals into intracellular responses (Howlett et al., 2010; Zou and Kumar, 2018).”

Bloomfield, M. A. P., Ashok, A. H., Volkow, N. D., and Howes, O. D. (2016). The effects of Δ9-tetrahydrocannabinol on the dopamine system. *Nature* 539, 369–377. doi: 10.1038/nature20153 was not cited in the article. The citation has now been inserted in the section **2.1 CB1 Receptor**, Paragraph 2 and should read:

“Therefore, the evidence outlined above indicates a key role of endocannabinoid and CB1 receptors in the dopamine system (Bloomfield et al., 2016).”

In the published article, there was an error: an unclear and vague statement was included.

A correction has been made to the section **Abstract**, Paragraph 1. This sentence previously stated:

“Dopamine is a hormone that is released by the adrenal gland and influences motor control and motivation.”

The corrected sentence appears below:

“Dopamine, a neurotransmitter and neuromodulator, is primarily released by dopaminergic neurons in the midbrain, particularly in the substantia nigra and the ventral tegmental area (VTA).”

In the published article, there was an error: an unclear and vague statement was included.

A correction has been made to the section **Abstract**, Paragraph 1. This sentence previously stated:

“The D1 family is known to play a role in motivation and motor control whereas the D2 family is known to affect attention and sleep.”

The corrected sentence appears below:

“The D1 family and D2 family work in conjunction, playing interconnected roles in reward processing and decision-making. The D1 family is composed of D1 and D5 receptors and primarily functions in motivation and motor control. In contrast, the D2 family, composed of D2, D3, and D4 receptors, affect attention and sleep.”

In the published article, there was an error: unclear wording was erroneously included.

A correction has been made to the section **Abstract**, Paragraph 1. This sentence previously stated:

“THC, a type of cannabinoid, can lead to feelings of euphoria, anxiety, fear, distrust, or panic. THC is known to affect dopamine in regions such as the anterior cingulate cortex (ACC), and plays a role in fundamental cognitive processes.”

The corrected sentence appears below:

“THC, a type of cannabinoid, can lead to feelings of euphoria, anxiety, fear, distrust, or panic, and modulates dopamine activity in several regions of the central nervous system.”

In the published article, there was an error: Unclear wording, a vague statement, potential content confusion and a reference mistake were erroneously included.

A correction has been made to the section **Introduction**, Paragraph 2. This sentence previously stated:

“Whereas CB1 receptors influence motor control, thinking, motor coordination, and related functions, CB2 receptors usually influence the gut, kidneys, pancreas, and related organs (Ashton and Glass, 2007). The CB1 receptors are located in different parts of the CNS such as the cerebral cortex, amygdala and hippocampus. The CB2 receptors are located in brainstem and hippocampal CA2/3 pyramidal neurons.”

The corrected sentence appears below:

“CB1 receptors are located in different parts of the CNS, including the cerebral cortex, amygdala, and hippocampus, and are associated with processes related to memory, learning, motor skills, and emotional responses (Arnau Busquets-Garcia et al., 2016). In contrast, CB2 receptors are primarily found in the peripheral nervous system (PNS), particularly in immune cells such as those in the spleen and macrophages, but they can also be present in the CNS (including the brainstem and CA2/3 pyramidal neurons of the hippocampus) and play a role in regulating immune functions (Turcotte et al., 2016).”

In the published article, there was an error: additional sentences need to be added for clarification.

A correction has been made to the section **Introduction**, Paragraph 2. This sentence previously stated:

“THC affects dopamine, a neurotransmitter needed for motor control, arousal and more.”

The corrected sentences appear below:

“THC affects dopamine, a neurotransmitter involved in motor control, arousal, and more. Specifically, within the central nervous system, THC regulates dopaminergic activity across several regions, including the dorsal and ventral striatum. Additionally, studies suggest that THC influences the anterior cingulate cortex (ACC), where dopamine plays a crucial role in regulating associated functions. This indicates that the effects of THC on the ACC may be mediated by its impact on dopamine signaling (Khani et al., 2014; Borgwardt et al., 2008; Bloomfield et al., 2016).”

In the published article, there was an error: unclear wording, vague statement and a lack of focus were erroneously included.

A correction has been made to the section **Introduction**, Paragraph 3. These sentences previously stated:

“Dopamine is a monoamine neurotransmitter, which is known to play a role in executive function, motor control, motivation, arousal, reinforcement, and reward. Dopamine release can be stimulated by conducting pleasurable behaviors. This causes the adrenal gland, which is located above the kidneys, to release dopamine.”

The corrected sentences appear below:

“Dopamine is a monoamine neurotransmitter, which plays a role in executive function, motor control, motivation, arousal, reinforcement, and reward. Central dopamine release can be stimulated by motivating stimuli and engaging in rewarding behaviors, such as nucleus accumbens. While dopamine is primarily produced in the brain by neurons in the substantia nigra and ventral tegmental area, the adrenal glands can also release dopamine into the bloodstream under certain conditions, such as stress. This causes the adrenal gland to release dopamine.”

In the published article, there was an error: unclear wording and a vague statement was erroneously included.

A correction has been made to the section **2 Dopamine**, Paragraph 1. This sentence previously stated:

“Dopamine is a hormone that is released from the adrenal gland which is controlled by the medulla.”

The corrected sentence appears below:

“Dopamine is a neurotransmitter and neuromodulator, which is synthesized in the ventral tegmental area (VTA), substantia nigra, and hypothalamus.”

In the published article, there was an error: unclear wording, a vague statement and a reference mistake were erroneously included.

A correction has been made to the section **2.1 CB1 Receptor**, Paragraph 2. This paragraph previously stated:

“CB1 receptors and endocannabinoid ligands anandamide and 2-arachidonoylglycerol, two major endocannabinoids, play a role in retrograde feedback activities on presynaptic glutamatergic and γ-aminobutyric acid (GABA) nerve terminals, and are known to be abundant in the dopaminergic pathways (Herkenham et al., 1991). CB1 antagonist rimonabant blocks anandamide (Solinas et al., 2006) and 2-AG's (De Luca et al., 2014) dopamine stimulation from the NAc shell. This shows that CB1 receptors are involved in mediating the dopaminergic effects of endocannabinoids. Biased signal transduction mechanisms from synaptic signaling in the midbrain contribute to the rewarding properties of THC and this is arbitrated by increased dopamine release and dopaminergic neuron firing. 2AG acts retroactively on CB1 receptors on glutamatergic and GABAergic terminals once it is synthesized by diacylglycerol lipase. CB1 receptors interfere with GABA inputs on midbrain dopamine cells (Lecca et al., 2011). CB1 receptors are also localized in glutamatergic terminals, which synapse on midbrain dopamine neurons (Marinelli et al., 2006), a place where endocannabinoids regulate excitation of retrograde suppression. Endocannabinoids, therefore, stabilize the activity of dopamine projections by regulating excitatory and inhibitory signaling.”

The corrected paragraph appears below:

“Dopaminergic pathways have many GABA(ergic) terminals which are modulated by CB1 receptors and endocannabinoids (such as anandamide and 2-AG). This modulation involves retrograde feedback, which is important for regulating synaptic transmission and maintaining balance between excitatory and inhibitory signals. This interaction also affects dopamine release, especially in areas such as the nucleus accumbens (NAc). When endocannabinoids act on CB1 receptors, they increase dopamine release. This mediates the dopaminergic effects of endocannabinoids, which is shown by CB1 antagonist rimonabant blocking the release of dopamine from the NAc shell that is stimulated by anandamide and 2-AG. Based on synaptic activity, the midbrain's response is altered, contributing to the rewarding effects of THC. This leads to increased dopamine release and firing of dopamine neurons. 2-AG, once produced, activates CB1 receptors on glutamate and GABA neurons. These receptors reduce GABA's effect on dopamine cells in the midbrain, which increases dopamine activity. This represents the role of CB1 receptors in dopamine regulation. Additionally, CB1 receptors play a role in balancing the excitatory and inhibitory signals which influence dopamine neurons, which is essential for stabilizing the activity of dopamine. Endocannabinoids modulate retrograde suppression in glutamate terminals, where CB1 receptors are localized and synapse with midbrain dopamine neurons. Therefore, the evidence outlined above indicates a key role of endocannabinoid and CB1 receptors in the dopamine system (Bloomfield et al., 2016).”

The original version of this article has been updated.

